# A Novel Self-Calibration Method and Experiment of MEMS Gyroscope Based on Virtual Coriolis Force

**DOI:** 10.3390/mi9070328

**Published:** 2018-06-28

**Authors:** Minruihong Wang, Huiliang Cao, Chong Shen, Jin Chai

**Affiliations:** Science and Technology on Electronic Test & Measurement Laboratory, North University of China, Tai Yuan 030051, China; wangmrh@outlook.com (M.W.); awesomecj95@outlook.com (J.C.)

**Keywords:** microelectromechanical systems (MEMS) vibratory gyroscopes, virtual Coriolis force, self-calibration, scale factor, force balance control

## Abstract

This paper proposes an effective method to calibrate the microelectromechanical systems (MEMS) vibratory gyroscope based on the virtual Coriolis force. This method utilizes a series of voltage signals to simulate the Coriolis force input, and the gyroscope output is monitored to obtain the scale factor characteristics of the gyroscopes. The scale factor and bias parameters of the gyroscope can be calibrated conveniently and efficiently in the sense-mode open loop. The calibration error of the scale factor based on the turntable and the virtual Coriolis force method is only 1.515%, which proves the correction of the method proposed in this paper. Meanwhile, the non-linearity and bias value of the turntable and the virtual Coriolis force method are 742 ppm and 42.04 mV and 3389 ppm and 0.66 mV, respectively.

## 1. Introduction

Microelectromechanical systems (MEMS) vibratory gyroscopes have had important applicable value in various fields, such as national economy, national defense, military, and navigation fields, for the past few years [1]. The scale factor represents a proportion between the measured voltage and the input angular rate. A MEMS vibratory gyroscope with more extensive applications must have both a better static characteristic and a better dynamic characteristic, and the scale factor is an important index to determine the two characteristics [2]. Therefore, it is necessary to calibrate the scale factor of the MEMS vibratory gyroscope.

The principle method by which MEMS vibratory gyroscopes respond to the external angular rate is the Coriolis force effect. When the substrate rotates, the vibrational mass block driven by the substrate rotates and generates the Coriolis force to measure the angular rate [3]. Therefore, if the vibrational amplitude of the oscillator is stable, the input angular rate will be directly proportional to the Coriolis force. MEMS vibratory gyroscopes are based on the oscillators and the detectors, which are respectively under the drive mode and the sense mode [4]. The optimum performance of the MEMS vibratory gyroscope depends on not only the mechanical structure, but also on the signal control loop, which plays an essential role [5], and the signal control loop is mainly composed of the drive circuit and the angular velocity demodulation circuit. Matching the drive signal frequency and the drive mode natural frequency is of great influence on the gyroscope’s detection sensitivity [6], so it is necessary to equalize the two frequencies to earn a high sensitivity. The Coriolis force in the detection direction is proportional to the vibration amplitude of the drive mode, so when the gyroscope is working, the stability of the vibration amplitude is a precondition to guarantee the precision of the gyroscope detection [7]. The drive mode usually adopts automatic gain control (AGC) and phase locked loop technology (PLL) for control. These methods make the frequency and the amplitude of the drive circuit stabilize, which can improve the performance of the gyroscope [8]. The higher sensitivity is caused by the higher *Q*-value in the gyroscope [9]. The MEMS vibratory gyroscope can get a higher *Q*-value under the vacuum package; meanwhile, its resolution, noise performance, and zero bias drift are obviously improved. It has been proven that getting the mode-matched under the high *Q*-value is an important approach to improve the precision of the MEMS vibratory gyroscope [10].

The scale factor and the bandwidth are two important indexes of MEMS vibratory gyroscopes that are usually obtained by angular vibrational tables and turntables [11]. The traditional calibration method requires a precise position reference to eliminate the Earth’s rotational acceleration and gravitational acceleration, so it will be greatly restricted in the extreme environment [12]. The errors of gyroscope assembly, turntables, angular vibrational tables, and the limitations of their performances also greatly restrict the accurate parameters of gyroscope performance [13]. The turntable cannot possibly provide a wide frequency response because of its performances, and it is very complicated to rely on the turntable to produce the Coriolis force effect in an actual system. So, some more convenient and more accurate method than the traditional method is needed to obtain the mechanical characteristic of the MEMS gyroscopes. Some literature has presented several calibration methods without using turntables and angular vibrational tables. Liu et al. presented a calibration method for a MEMS gyroscope based on the output of a magnetic sensor. But, the method can work only when the magnet is not parallel to the rotation axis of the MEMS gyroscope, and an additional magnetic sensor is needed [14]. Alexander et al. used the gravitational force on the gyroscope detection mass to generate a reference to the Coriolis force in order to obtain the scale factor of the gyroscope [15]. In [16], a calibration method based on the virtual Coriolis force was used to measure the frequency response of the MEMS structure, but this theory and the experiment results did not reflect the detailed nonlinear characteristic of the scale factor. Trusov et al. made a modulated square-wave as the virtual rate signal to detect and calibrate the scale factor of a closed-loop operated vibratory gyroscope [17]. Prikhodko et al. presented a method to reduce the angle-dependent bias drifts of MEMS rate integrating gyroscopes via virtual rotation using the gyroscopes’ control electrodes along with dynamic mismatch controls [18].

This study presents a self-calibration method that uses an electrostatic signal, which is applied to the force feedback comb figures to generate the virtual Coriolis force in order to calibrate the performance of MEMS vibratory gyroscopes. The experimental results show that the method is feasible. The system in this paper can calibrate the scale factor of the MEMS gyroscope effectively.

## 2. Principle Analysis of MEMS Gyroscopes

**Figure 1:**
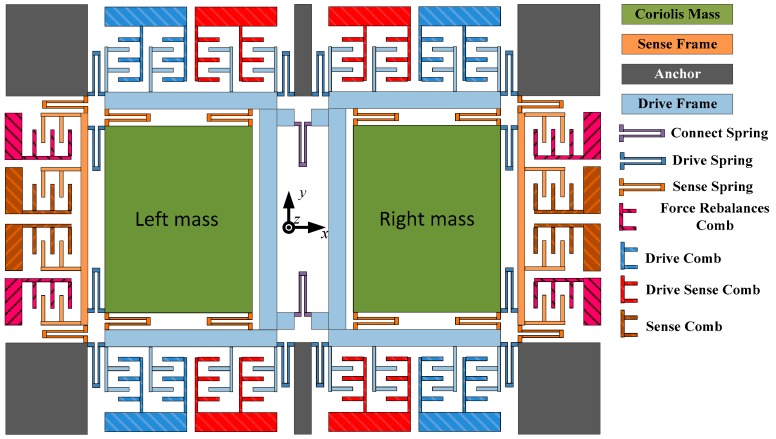
The mechanical structure of dual-mass microelectromechanical systems (MEMS) gyroscopes.

The working principle of the MEMS vibratory gyroscope can be equivalent to a second-order mass-damping-spring system; its drive and sense shafts are *x*- and *y*-axis, and the angular rate is input along its sensitive shaft, the z-axis. The working condition of the MEMS vibratory gyroscope is that the drive mode makes constant vibrational amplitude in the *x*-axis. When the drive mode stimulating frequency is equal to its resonant frequency, drive mode obtains the maximum amplitude. The angular rate is inputted in the *z*-axis, and then, the *y*-axis produces the Coriolis force. So, the displacement is detected by the *y*-axis, and the angular rate can be obtained through calculations. Figure 1 is the structure of the dual-mass MEMS gyroscope. The tuning fork is the drive mode of this structure, and the two coupled drive masses, named left mass and right mass, respectively, are connected by two purple U-shaped springs, named connect spring, in the center of the structure. Two coupled sense frames are connected with the Coriolis masses respectively through several drive springs on the *x*-axis [19].

**Figure 2:**
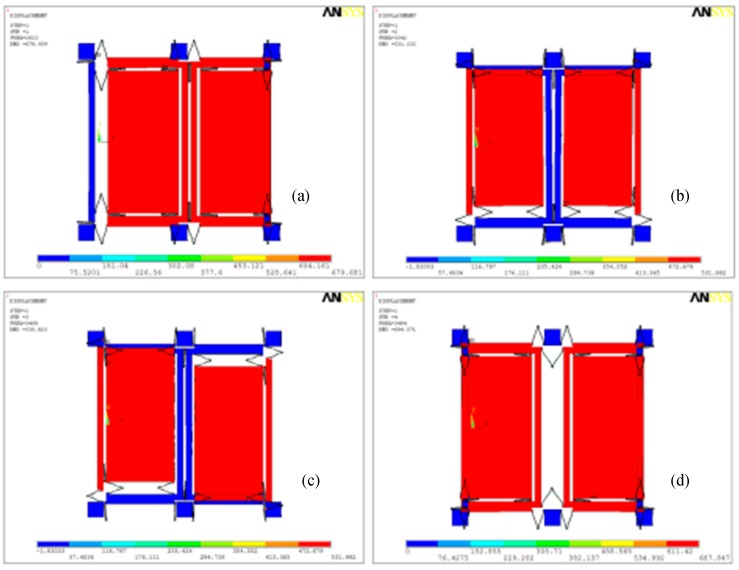
(**a**) The first mode: drive in phase mode $\omega_{x1}=2623\times 2\pi \;\mathrm{rad}/s$; (**b**) The second mode: sense in phase mode $\omega_{y1}=3342\times 2\pi \;\mathrm{rad}/s$; (**c**) The third mode: sense anti-phase mode $\omega_{y2}=-3468\times 2\pi \;\mathrm{rad}/s$; and (**d**) The fourth mode: drive anti-phase mode $\omega_{x2}=3484\times 2\pi \;\mathrm{rad}/s$.

**Figure 3:**
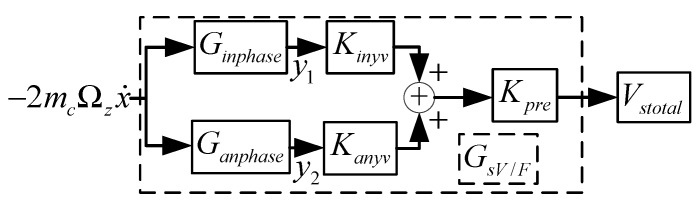
The real working model schematic diagram of the sense mode.

The first four order modes are simulated in ANSYS simulation software (12.0, Ansys, Inc., Canonsburg, PA, USA), as shown in Figure 2. Where $\omega_{x1},\omega_{x2},\omega_{y1},\;$ and $\omega_{y2}$ are the first four order modes’ resonant frequencies of the dual-mass MEMS gyroscope. There is a large gap, which is greater than 1000 Hz between the frequencies of the drive in phase and anti-phase modes, and the *Q*-value of the drive mode in the anti-phase named $Q_{x2}$ is greater than 2000 (calculated from the experiment results). So, the drive mode can be considered as working in the anti-phase mode. The real working mode of the sense mode is formed by the second and third modes and is shown in Figure 3 [2].

Considering the real working method of the drive and sense mode, in the ideal condition, the structural motion equation of the MEMS gyroscope is expressed as follows [2]:

(1) $$m\ddot{D}+c\dot{D}+kD=F$$

where the mass matrix $m=\left[\begin{array}{c} m_{x}\\ m_{y}\\ m_{y} \end{array}\right]$, the displacement matrix $D=\left[\begin{array}{c} x\\ y_{1}\\ y_{2} \end{array}\right]$, the stiffness matrix $k=\left[\begin{array}{ccc} \omega_{x2}^{2}m_{x} & 0 & 0\\ 0 & \omega_{y1}^{2}m_{y} & 0\\ 0 & 0 & \omega_{y2}^{2}m_{y} \end{array}\right]$, the damping matrix $c=\left[\begin{array}{ccc} \frac{\omega_{x2}m_{x}}{Q_{x2}} & 0 & 0\\ 0 & \frac{\omega_{y1}m_{y}}{Q_{y1}} & 0\\ 0 & 0 & \frac{\omega_{y2}m_{y}}{Q_{y2}} \end{array}\right]$, and the external matrix $F=\left[\begin{array}{c} F_{d}sin\left(\omega_{d}t\right)\\ -2m_{c}\Omega_{z}\dot{x}\\ -2m_{c}\Omega_{z}\dot{x} \end{array}\right]$. $m_{x}$ is the equivalent mass of the drive mass; x is the displacement of the drive mode. The drive modes in the phase and in the anti-phase have displacements respectively named $y_{1}$ and $y_{2}$ and *Q*-values respectively named $Q_{y1}$ and $Q_{y2}$. $\Omega_{z}$ is the input of the angular rate; $m_{y}$ is the mass of the sense mode, which is approximately equal to the Coriolis mass $m_{c}$; $F_{d}$ is the stimulating of the drive mode; and $\omega_{d}$ is the frequency of the drive mode. Because the displacement of the sense mode $y=y_{1}+y_{2}$, x(t) can be expressed as follows:

(2) $$\begin{array}{l} x\left(t\right)\\ =\frac{F_{d}/m_{x}}{\sqrt{{\left(\omega_{x2}^{2}-\omega_{d}^{2}\right)}^{2}+\omega_{x2}^{2}\omega_{d}^{2}/Q_{x2}^{2}}}\sin\left(\omega_{d}t+\varphi_{x2}\right)\\ +\frac{F_{d}\omega_{x2}\omega_{d}/m_{x}Q_{x}}{{\left(\omega_{x2}^{2}-\omega_{d}^{2}\right)}^{2}+\omega_{x2}^{2}\omega_{d}^{2}/Q_{x2}^{2}}e^{-\frac{\omega_{x2}}{2Q_{x2}}t}\;\cos\left(\sqrt{1-1/4Q_{x2}^{2}}\omega_{x2}t\right)\\ +\frac{F_{d}\omega_{d}\left(\omega_{x2}^{2}/Q_{x2}^{2}+\omega_{d}^{2}-\omega_{x2}^{2}\right)/m_{x}}{\omega_{x2}\sqrt{1-1/4Q_{x2}^{2}}\left[{\left(\omega_{x2}^{2}-\omega_{d}^{2}\right)}^{2}+\omega_{x2}^{2}\omega_{d}^{2}/Q_{x2}^{2}\right]}e^{-\frac{\omega_{x2}}{2Q_{x2}}t}\sin\left(\sqrt{1-1/4Q_{x2}^{2}}\omega_{x2}t\right) \end{array}$$

And, $y_{1,2}\left(t\right)$ can be expressed as follows:

(3) $$\begin{array}{l} y_{1,2}\left(t\right)\\ =\frac{F_{c}}{\sqrt{{\left(\omega_{y1,2}^{2}-\omega_{d}^{2}\right)}^{2}+\omega_{y1,2}^{2}\omega_{d}^{2}/Q_{y1,2}^{2}}}\sin\left(\omega_{d}t+\varphi_{x2}+\frac{\pi }{2}+\varphi_{y1,2}\right)\\ -\frac{F_{c}\left[\omega_{y1,2}\omega_{d}\sin\varphi_{x2}/Q_{y1,2}+\left(\omega_{y1,2}^{2}-\omega_{d}^{2}\right)\cos\varphi_{x2}\right]}{{\left(\omega_{y1,2}^{2}-\omega_{d}^{2}\right)}^{2}+\omega_{y1,2}^{2}\omega_{d}^{2}/Q_{y1,2}^{2}}e^{-\frac{\omega_{y1,2}}{2Q_{y1,2}}t}\cos\left(\sqrt{1-1/4Q_{y1,2}^{2}}\omega_{y1,2}t\right)\\ +\frac{F_{c}\left[\omega_{y1,2}\left(\omega_{y1,2}^{2}-3\omega_{d}^{2}\right)\cos\varphi_{x2}/2Q_{y1,2}+\omega_{d}\left(\omega_{y1,2}^{2}/2Q_{y1,2}^{2}+\omega_{y1,2}^{2}-\omega_{d}^{2}\right)\sin\varphi_{x2}\right]}{\omega_{y1,2}\sqrt{1-1/4Q_{y1,2}^{2}}\left[{\left(\omega_{y1,2}^{2}-\omega_{d}^{2}\right)}^{2}+\omega_{y1,2}^{2}\omega_{d}^{2}/Q_{y1,2}^{2}\right]}e^{-\frac{\omega_{y1,2}}{2Q_{y1,2}}t}\sin\left(\sqrt{1-1/4Q_{y1,2}^{2}}\omega_{y1,2}t\right) \end{array}$$

where $\;\varphi_{x2}=-tg^{-1}\left[\frac{\omega_{x2}\omega_{d}}{Q_{x2}\left(\omega_{x2}^{2}\;-\;\omega_{d}^{2}\right)}\right],\;F_{c}=\frac{-2\Omega_{z}\omega_{d}F_{d}}{m_{x}\sqrt{{\left(\omega_{x2}^{2}-\omega_{d}^{2}\right)}^{2}\;+\;\omega_{x2}^{2}\omega_{d}^{2}/Q_{x2}^{2}}}$ and $\varphi_{y1,2}=-tg^{-1}\left[\frac{\omega_{y1,2}\omega_{d}}{Q_{y1,2}\left(\omega_{y1,2}^{2}\;-\;\omega_{d}^{2}\right)}\right]$. The above equations indicate that the movements of the sense mode and the drive mode are the compound motions of the stable vibration and the attenuation vibration. The drive mode employs closed-loop, and the drive mode $\;\omega_{d}=\omega_{x2}$, working with the stable-amplitude vibration and the attenuation vibration, can be ignored. Then, the formula *x(t)* can be simplified as follows:

(4) $$\;x\left(t\right)=\frac{F_{d}Q_{x2}}{m_{x}\omega_{d}^{2}}\cos\left(\omega_{d}t\right)=A_{x}\cos\left(\omega_{d}t\right)$$

The stable vibration characteristic can be derived from the following Equation:

(5) $$\;y_{1,2}\left(t\right)=\frac{-2\Omega_{z}F_{d}Q_{x2}\sin\left(\omega_{d}t\right)}{m_{x}\omega_{d}\sqrt{{\left(\omega_{y1,2}^{2}-\omega_{d}^{2}\right)}^{2}+\omega_{y1,2}^{2}\omega_{d}^{2}/Q_{y1,2}^{2}}}=A_{y1,2}\sin\left(\omega_{d}t\right)$$

And the mechanical sensitivity is expressed as follows:

(6) $$\;S_{me}=\frac{A_{y1}+A_{y2}}{\Omega_{z}}\approx \frac{-F_{d}Q_{x}}{m_{x}\omega_{d}^{2}}\left(\frac{1}{\omega_{y1}-\omega_{x2}}+\frac{1}{\omega_{y2}-\omega_{x2}}\right)=-A_{x}\left(\frac{1}{\Delta \omega_{1}}+\frac{1}{\Delta \omega_{2}}\right)$$

The vibration amplitude of the drive mode and the frequency gaps between the drive working mode and the sense mode, which include the second mode and the third mode, determine the mechanical sensitivity of the dual-mass sense-coupled structure. The one near the fourth mode is a dominant element. That is, the anti-phase sense mode determines the structural mechanical sensitivity. Figure 3 is the schematic diagram of the sense mode. $G_{inphase\;}$ and $K_{inyv}$ are the transform function and the displace-voltage transform parameters of the sense in phase mode, respectively. Similarly $G_{anphase\;}$ and $K_{anyv}$ are the transform function and the displace-voltage transform parameters of the sense anti-phase mode, respectively. $K_{pre}$ is the pre-amplifier, and $V_{stotal}$ is the output of the sense mode. Then, $G_{sV/F}$ can be expressed as follows:

(7) $$\;G_{sV/F}=\left(G_{inphase}K_{inyv}+G_{anphase}K_{anyv}\right)K_{pre}$$

In the above formula, $G_{inphase}=\frac{1}{m_{y}}\frac{1}{s^{2}\;+\;\frac{\omega_{y1}}{Q_{y1}}s\;+\;\omega_{y1}^{2}}$ and $G_{anphase}=\frac{1}{m_{y}}\frac{1}{s^{2}\;+\;\frac{\omega_{y2}}{Q_{y2}}s\;+\;\omega_{y2}^{2}}$.

## 3. Theoretical Analysis of Virtual Coriolis Force

The basic principle of the MEMS vibration gyroscope is based on the Coriolis coupling effect. The Coriolis force transfers the energy from the drive mode to the sense mode [20,21].The frequency response of the sense mode is the Coriolis force effect added to the movable mass block. Under the condition that the Coriolis force response cannot be obtained directly, the virtual force with the same characteristics as the Coriolis force is added on the gyroscope’s mass block to detect its response. Then, the response to the Coriolis force is calibrated indirectly after comparing the relationship between the virtual Coriolis force and the actual Coriolis force.

In this work, the gyroscope works in sense-mode open-loop mode, at this time, the mechanical sensitivity and the bandwidth are contradictory, and the bandwidth is 0.54Δω.

**Figure 4:**
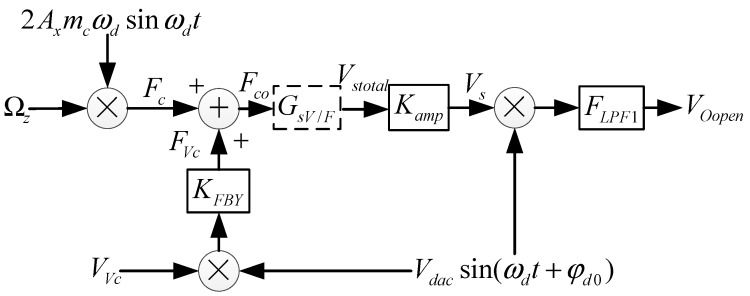
The virtual Coriolis force system and gyroscope open-loop signal flow chart.

The virtual Coriolis force system is shown in Figure 4, which is a signal flow chart of the angular rate in the gyro system. The phase-sensitive demodulation method is employed to pick up the amplitude of the Coriolis in-phase signal, which is related to the Coriolis force. $F_{LPF}$ is the transfer function of the low-pass filer, and $K_{amp}$ is the transfer function of the amplifier. $F_{c}$ is the real Coriolis force generated by $\Omega_{Z}$, $F_{Vc}$ is the virtual Coriolis force generated by the virtual Coriolis force voltage $V_{Vc}$, and $F_{Vc}$ is the substitution of $F_{c}$ and $F_{Vc}$. $K_{FBY}$ is the voltage-electrostatic transform coefficient of feedback combs. Let the coefficient from the angular rate to the real Coriolis force be named the external input angular rate, and the vibration rate of the drive mode is $\;A_{d}\omega_{d}cos\omega_{d}t$. Then, the Coriolis force can be expressed as 

(8) $$\;\{\begin{array}{c} F_{c}=2m_{y}A_{x}\omega_{d}\Omega_{Z}sin\left(\omega_{d}t\right)\\ F_{Vc}=V_{Vc}K_{FBY}V_{dac}sin\left(\omega_{d}t+\varphi_{d0}\right)\\ F_{co}=F_{Vc}+F_{c}\\ V_{s}=F_{co}G_{sV/F}K_{amp} \end{array}$$

In the formula,$\;V_{dac}$ is the amplitude of demodulation signal.

Then, the output signal of the gyroscope can be expressed as:

(9) $$\;V_{Oopen}=V_{dac}\sin\left(\omega_{d}t+\varphi_{d0}\right)V_{s}F_{LPF1}$$

Next, input Equation (8) to Equation (9) and make $m_{y}\approx m_{c}$, and there are two situations as follows:

Situation 1: $V_{Vc}=0\;V$, and this is the open-loop condition. The gyroscope works with the real Coriolis force generated by the angular rate input signal $\Omega_{z}$. After Laplace transformation, we can get the output signal of the gyroscope structure as follows:

(10) $$V_{stotal}\left(s\right){|}_{V_{Vc=0}}=A_{x}\omega_{d}K_{pre}\left(\Omega_{z}\left(s-j\omega_{d}\right)+\Omega_{z}\left(s+j\omega_{d}\right)\right)\left[\frac{K_{inyv}}{s^{2}+\frac{\omega_{y1}}{Q_{y1}}s+\omega_{y1}^{2}}+\frac{K_{anyv}}{s^{2}+\frac{\omega_{y2}}{Q_{y2}}s+\omega_{y2}^{2}}\right]$$

Then, the output signal of gyroscope is as follows:

(11) $$V_{Oopen}\left(s\right){|}_{V_{Vc=0}}=\;\frac{1}{2}A_{x}\omega_{d}V_{dac}K_{pre}F_{LPF1}\left(s\right)\;\left\{\begin{array}{c} \left[\frac{\left[\Omega_{z}\left(s\right)\;+{\;\Omega }_{z}\left(s\;+\;2j\omega_{d}\right)\right]e^{-j\varphi_{d0}}K_{inyv}}{{\left(s\;+\;j\omega_{d}\right)}^{2}\;+\;\frac{\omega_{y1}}{Q_{y1}}\left(s\;+\;j\omega_{d}\right)\;+\;\omega_{y1}^{2}}\;+\;\frac{\left[\Omega_{z}\left(s\right)\;+\;\Omega_{z}\left(s\;+\;2j\omega_{d}\right)\right]e^{-j\varphi_{d0}}K_{anyv}}{{\left(s\;+\;j\omega_{d}\right)}^{2}\;+\frac{\omega_{y2}}{\;Q_{y2}}\left(s\;+\;j\omega_{d}\right)\;+\;\omega_{y2}^{2}}\right]\\ +\left[\frac{\left[\Omega_{z}\left(s\;-\;2j\omega_{d}\right)\;+{\;\Omega }_{z}\left(s\right)\right]e^{j\varphi_{d0}}K_{inyv}}{{\left(s\;-\;j\omega_{d}\right)}^{2}\;+\;\frac{\omega_{y1}}{Q_{y1}}\left(s\;-\;j\omega_{d}\right)\;+\;\omega_{y1}^{2}}+\frac{\left[\Omega_{z}\left(s\;-\;2j\omega_{d}\right)\;+{\;\Omega }_{z}\left(s\right)\right]e^{j\varphi_{d0}}K_{anyv}}{{\left(s-j\omega_{d}\right)}^{2}\;+\;\frac{\omega_{y2}}{Q_{y2}}\left(s\;-\;j\omega_{d}\right)\;+\;\omega_{y2}^{2}}\right] \end{array}\right\}$$

Considering the low-pass filter effect of $F_{LPF1}\left(s\right)$, the cut-off frequency is 100 Hz, and the double frequency ($2\omega_{d}$) components are filtered out. The scale factor of the MEMS gyroscope sense-mode system open loop can be expressed as:

(12) $$\left|\frac{V_{Oopen}\left(s\right){|}_{V_{Vc=0}}}{\Omega_{z}\left(s\right)}\right|=\left|\frac{1}{2}A_{x}\omega_{d}V_{dac}K_{pre}F_{LPF1}\left(s\right)G_{equal}\left(s\right)\right|$$

where, $G_{equal}\left(s\right)=\{\frac{K_{inyv}\left(s^{2}\;+\;\frac{\omega_{y1}}{Q_{y1}}s\;+\;\omega_{y1}^{2}-\omega_{d}^{2}\right)}{{\left(s^{2}\;+\;\frac{\omega_{y1}}{Q_{y1}}s\;+\;\omega_{y1}^{2}\;-\;\omega_{d}^{2}\right)}^{2}+\;{\left(2s\omega_{d\;}+\;\frac{\omega_{y1}}{Q_{y1}}\omega_{d}\right)}^{2}}+\;\frac{K_{anyv}\left(s^{2}\;+\;\frac{\omega_{y2}}{Q_{y2}}s\;+\;\omega_{y2}^{2}\;-\;\omega_{d}^{2}\right)}{{\left(s^{2}\;+\;\frac{\omega_{y2}}{Q_{y2}}s\;+\;\omega_{y2}^{2}\;-\;\omega_{d}^{2}\right)}^{2}\;+\;{\left(2s\omega_{d}\;+\;\frac{\omega_{y2}}{Q_{y2}}\omega_{d}\right)}^{2}}\}$

Situation 2: $\Omega_{z}=0\;{}^{\circ}/s$, and this is the virtual Coriolis force calibration condition. The gyroscope works with the virtual Coriolis force generated by the electrostatic force $V_{Vc}$. After Laplace transformation, we can get the output signal of the gyroscope structure as follows:

(13) $$V_{stotal}\left(s\right){|}_{\Omega_{z}=0}=\frac{K_{pre}V_{dac}K_{FBY}}{2m_{y}}\left(V_{Vc}\left(s-j\omega_{d}\right)+V_{Vc}\left(s+j\omega_{d}\right)\right)\left[\frac{K_{inyv}}{s^{2}\;+\;\;\frac{\omega_{y1}}{Q_{y1}}s\;+\;\omega_{y1}^{2}}\;+\;\frac{K_{anyv}}{s^{2}\;+\;\frac{\omega_{y2}}{Q_{y2}}s\;+\;\omega_{y2}^{2}}\right].$$

Then, the output signal of the gyroscope under the virtual Coriolis force calibration condition is as follows:

(14) $$V_{Oopen}\left(s\right){|}_{\Omega_{z}=0}=\;\frac{1}{4}\frac{K_{pre}K_{FBY}V_{dac}^{2}F_{LPF1}\left(s\right)}{m_{y}}\;\left\{\begin{array}{c} \left[\frac{\left[V_{Vc}\left(s\right)\;+\;V_{Vc}\left(s\;+\;2j\omega_{d}\right)\right]K_{inyv}}{{\left(s\;+\;j\omega_{d}\right)}^{2}\;+\;\frac{\omega_{y1}}{Q_{y1}}\left(s\;+\;j\omega_{d}\right)\;+\;\omega_{y1}^{2}}\;+\;\frac{\left[V_{Vc}\left(s\right)\;+{\;V}_{Vc}\left(s\;+\;2j\omega_{d}\right)\right]K_{anyv}}{{\left(s\;+\;j\omega_{d}\right)}^{2}\;+\;\frac{\omega_{y2}}{Q_{y2}}\left(s\;+\;j\omega_{d}\right)\;+\;\omega_{y2}^{2}}\right]\\ +\left[\frac{\left[V_{Vc}\left(s\;-2j\omega_{d}\right)\;+\;V_{Vc}\left(s\right)\right]K_{inyv}}{{\left(s\;-\;j\omega_{d}\right)}^{2}\;+\;\frac{\omega_{y1}}{Q_{y1}}\left(s\;-\;j\omega_{d}\right)\;+\;\omega_{y1}^{2}}\;+\;\frac{\left[V_{Vc}\left(s\;-\;2j\omega_{d}\right)\;+\;V_{Vc}\left(s\right)\right]K_{anyv}}{{\left(s\;-\;j\omega_{d}\right)}^{2}\;+\;\frac{\omega_{y2}}{Q_{y2}}\left(s\;-\;j\omega_{d}\right)\;+\;\omega_{y2}^{2}}\right] \end{array}\right\}$$

Also, considering the low-pass filter effect of $F_{LLPF1}\left(s\right)$ (the cut-off frequency is 100 Hz), and the scale factor of the MEMS gyroscope sense-mode system under the virtual Coriolis force calibration condition can be expressed as follows:

(15) $$\;\left|\frac{V_{Oopen}\left(s\right){|}_{\Omega_{z}=0}}{V_{Vc}\left(s\right)}\right|=\left|\frac{1}{4}\frac{K_{pre}K_{FBY}V_{dac}^{2}F_{LPF1}\left(s\right)}{m_{y}}G_{equal}\left(s\right)\right|$$

Comparing Equations (12) and (15), it is obvious that different $\Omega_{z}$ can be substituted by varying $V_{Vc}$ voltage, and their relationship is as follows:

(16) $$\Omega_{z}=\frac{K_{FBY}V_{dac}}{2A_{x}\omega_{d}m_{y}}V_{Vc}\;=K_{Vc}V_{Vc}\;$$

## 4. System Building and Testing

### 4.1. System Building

**Figure 5:**
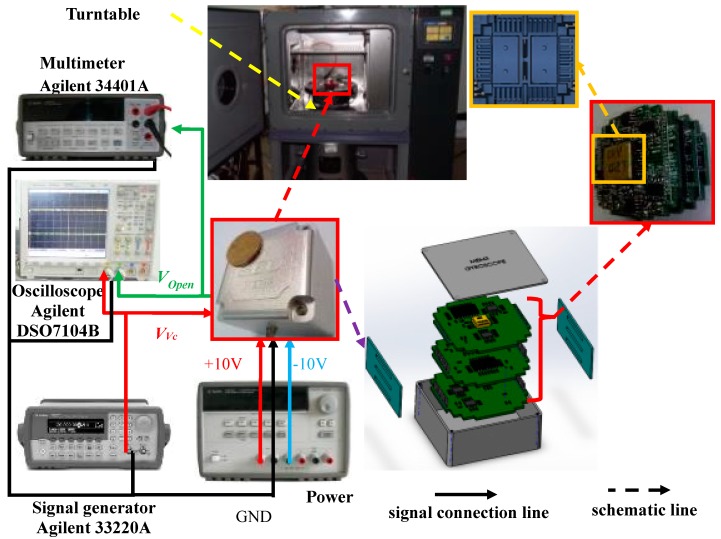
Circuit of dual-mass line vibrating gyroscope.

In order to verify the self-calibration method of the virtual Coriolis force, the experiment was arranged, and the equipment and gyroscope sample are shown in Figure 5. The power (Agilent E3631A, Keysight Technologies, Santa Rosa, CA, USA), provides ± 10 V direct current (DC) voltage and ground (GND); the oscilloscope (Agilent DSO7104B, Keysight Technologies) and multimeter (Agilent 34401A, Keysight Technologies) are employed to observe and measure the signal phase and amplitude. The virtual angular rate and the virtual vibration rate are respectively generated by a signal generator (Agilent 33220A, Keysight Technologies), which ensures the voltage signal has high precision and stability. The turntable is utilized to provide the real angular rate input $\Omega_{z}$ to test the scale factor of the MEMS gyroscope.

The structure is in a vacuumed package. The feedback combs (adopting a slide-film comb) are utilized to generate electronic force, which is performed as the virtual Coriolis force. The stimulated signal applied on feedback combs has the same frequency and phase as the Coriolis force. The whole control loop is a pure analog circuit. The monitoring circuit is arranged in three printed circuit boards (PCBs), their electronic signal and mechanical structure connect with each other through metal pins. The first PCB connects the structure chip, is the interface, and processes the weak signals. The second PCB is the drive closed loop, and the drive closed loop employs AGC technology and ensures the loop works stably. The third PCB is the sense loop with the virtual Coriolis force calibration circuit. The PCBs are packaged by rubber pad and then put into the metal shell. The rubber pad protects the PCBs and the structure chip from the shock and vibration. The metal shell is connected with the “GND” signal to provide an electromagnetic shield.

### 4.2. Experimental Test

The test circuit shown in Figure 5 and the packed dual-mass liner vibratory gyroscope can verify the method by which the virtual Coriolis force replaces the actual Coriolis force. The output signal value is collected with 1 Hz sampling frequency and is recorded with 10 second-average value. The experiment process is as follows:

Firstly, the gyroscope is fixed onto the turntable stably, and it is made sure that the $V_{Vc}=0\;V$ and $\Omega_{z}=10{}^{\circ}/s$. The output of gyroscope is recorded and named $\Omega_{z10}$.

Secondly, $\Omega_{z}=0{}^{\circ}/s,$ and $V_{Vc}$ is configured. The output of gyroscope should be equal to $\Omega_{z10}$, and the voltage should be $V_{Vc10}$. Then, the coefficient $K_{Vc}$ in Equation (16) is confirmed.

Thirdly, $\Omega_{z}$ is made to equal different values and the gyroscope is calibrated with the turntable. $V_{Vc}=0\;V$ during this process. The values are shown in Table 1.

Fourthly, $V_{Vc}$ is configured to equal different values to simulate the corresponding $\Omega_{z}$ value, and the output value is recorded, as shown in Table 1.

**Figure 6:**
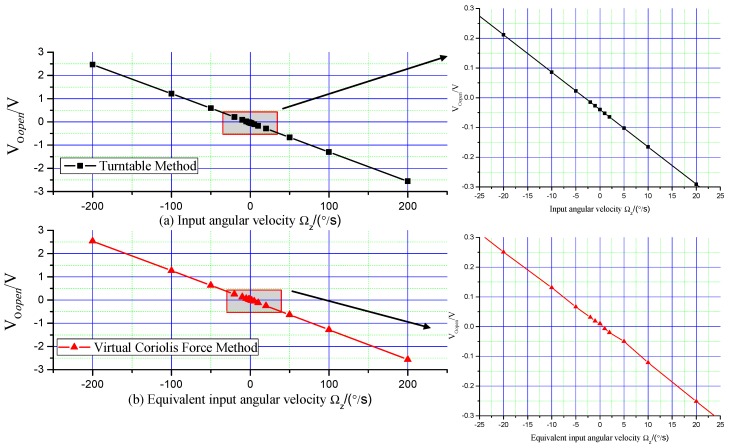
Calibration results of turntable method and the virtual Coriolis force method.

The curve is shown in Figure 6a. The parameters are analyzed by least square method, and the linear fit equation of the turntable calibration method is shown below. The value of non-linearity is 742 ppm.

(17) $$\;V_{Oopen}{|}_{\Omega_{z}}=-0.01254\Omega_{Z}-0.04204$$

The virtual Coriolis force calibration method values are analyzed by least square method, and the curve is drawn in Figure 6b. The linear fit equation is shown as below. The value of non-linearity is 3389 ppm.

(18) $$\;V_{Oopen}{|}_{V_{Vc}}=-0.01273\Omega_{Z}+0.00066$$

From the curves in Figure 6 and the analysis results above, it can be concluded that the scale factor value calibration error between the turntable and the virtual Coriolis force method is 1.515%. The non-linearity of the turntable method is better; this paper considers this reasoning from two aspects as follows:

Error 1: The voltage-electrostatic transform coefficient of feedback combs $K_{FBY}$ of two masses are not the same (because of the process error). The same virtual Coriolis force is applied on two masses, so the virtual Coriolis forces are different on two masses.

Error 2: The calculation and calibration process of the coefficient $K_{Vc}$ in Equation (16) generates some error, and this should be investigated in the future.

**Figure 7:**
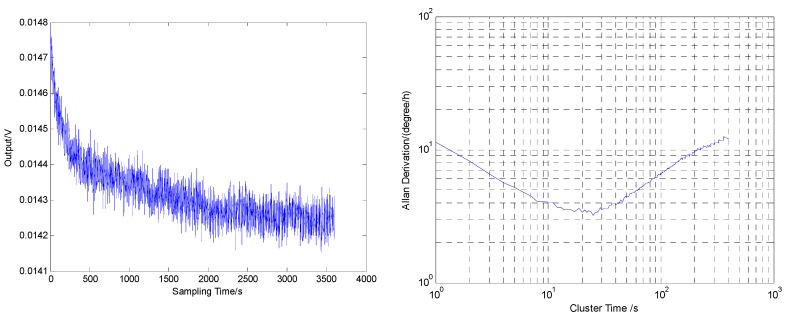
Output signal and Allan derivation of the output signal.

The quality factor of the gyroscope drive and sense mode are 1395 and 1224, respectively, at 20 °C. The resonant frequencies of the drive and sense mode are 3488.9 Hz and 3464.1 Hz, respectively. The output signal of the gyroscope collected with the sampling rate is 1 Hz, and 3600 points are picked up. The angular rate walk of the gyroscope is 0.21°/$\sqrt{h}$, and the bias stability is 3.7°/h, as shown in Figure 7 (calculated by the Allan derivation method) [22].

## 5. Conclusions

This paper represents a self-calibration method based on the virtual Coriolis force method. This method generates electrostatic force signals through force feedback combs to stimulate the sense-mode to calibrate the performance of the MEMS vibratory gyroscope. This method calibrates the gyroscope without the turntable and makes the calibration process more convenient. The experimental results show that the scale factor calibrated through the virtual Coriolis force method is similar to the turntable method (the error is only 1.515%). The experiment result verifies the virtual Coriolis force method proposed in this paper.

## Figures and Tables

**Table 1:** Dual-mass microelectromechanical systems (MEMS) gyroscope scale factor calibration experiment at room temperature (20 °C).

$\;\Omega_{z}/\left({}^{\circ}/s\right)$	MEMS Gyroscope Output/V	
	Turntable Calibration Method	Virtual Coriolis Force Calibration Method
200	−2.5536	−2.5626
100	−1.2970	−1.2791
50	−0.6690	−0.6352
20	−0.2925	−0.2517
10	−0.1672	−0.1215
5	−0.1043	−0.0496
2	−0.0666	−0.0199
1	−0.0541	−0.0074
0	−0.0418	0.0099
−1	−0.0290	0.0187
−2	−0.0164	0.0313
−5	0.0214	0.0661
−10	0.0842	0.1309
−20	0.2101	0.2509
−50	0.5862	0.6300
−100	1.2127	1.2645
−200	2.4622	2.5359
